# ATAC-clock: An aging clock based on chromatin accessibility

**DOI:** 10.1007/s11357-023-00986-0

**Published:** 2023-11-04

**Authors:** Francesco Morandini, Cheyenne Rechsteiner, Kevin Perez, Viviane Praz, Guillermo Lopez Garcia, Laura C. Hinte, Ferdinand von Meyenn, Alejandro Ocampo

**Affiliations:** 1 Department of Biomedical Sciences, University of Lausanne, Lausanne, Switzerland; 2 EPITERNA SA, Route de la Corniche 5, Epalinges, Switzerland; 3 Departamento de Lenguajes y Ciencias de la Computación, Universidad de Málaga, Málaga, Spain; 4 Department of Health Sciences and Technology, ETH Zurich, Zurich, Switzerland

**Keywords:** Aging, Epigenetic clock, Chromatin accessibility, ATAC sequencing, Biomarker

## Abstract

The establishment of aging clocks highlighted the strong link between changes in DNA methylation and aging. Yet, it is not known if other epigenetic features could be used to predict age accurately. Furthermore, previous studies have observed a lack of effect of age-related changes in DNA methylation on gene expression, putting the interpretability of DNA methylation-based aging clocks into question. In this study, we explore the use of chromatin accessibility to construct aging clocks. We collected blood from 159 human donors and generated chromatin accessibility, transcriptomic, and cell composition data. We investigated how chromatin accessibility changes during aging and constructed a novel aging clock with a median absolute error of 5.27 years. The changes in chromatin accessibility used by the clock were strongly related to transcriptomic alterations, aiding clock interpretation. We additionally show that our chromatin accessibility clock performs significantly better than a transcriptomic clock trained on matched samples. In conclusion, we demonstrate that the clock relies on cell-intrinsic chromatin accessibility alterations rather than changes in cell composition. Further, we present a new approach to construct epigenetic aging clocks based on chromatin accessibility, which bear a direct link to age-related transcriptional alterations, but which allow for more accurate age predictions than transcriptomic clocks.

**Supplementary Information:**

The online version contains supplementary material available at 10.1007/s11357-023-00986-0.

## Background

Aging is a biological process that is characterized by a progressive loss of physiological integrity on multiple biological scales and increased vulnerability to disease and death [1]. Current global demographic trends toward an aged population highlight the importance of studying aging to understand its dynamics and mitigate its role as a driver of diseases late in life [2].

Epigenetics perturbations are a hallmark of aging [1, 3]. In particular, the development of DNA-methylation-based aging clocks has shown that changes in methylation occur throughout life and can be used to accurately predict age [4–9]. Since the discovery of methylation clocks, the field has shown that clocks can be built from other biological signals, such as transcriptomic or proteomic profiles [10–17]. Despite exploration of these different signals, epigenetic clocks have exclusively used DNA methylation, thus far, partially due to data availability. Nonetheless, epigenetic regulation encompasses many mechanisms beyond DNA methylation [3]. Whether these different layers of epigenetic regulation can be used to predict age remains an open question. Additionally, because methylation of individual CpGs correlates poorly with transcription of the downstream genes, it can be difficult to interpret what biological processes correspond to methylation features used by clocks [18]. For these reasons, we sought to create an aging clock based on chromatin accessibility. Chromatin accessibility integrates the effect of multiple epigenetic mechanisms and therefore provides a more comprehensive description of chromatin states than DNA methylation [19]. Previous studies have observed age-related changes in chromatin accessibility in multiple organisms [20–23]. Moreover, the heterochromatin loss theory of aging stems from observations of global de-repression of chromatin during aging [24, 25]. We envision that these age-related changes will allow the construction of a clock and expand our understanding of epigenetic dysregulation.

In this study, we generated chromatin accessibility and transcriptomic profiles from human blood samples spanning a broad range of ages using ATAC-seq [26, 27] and RNA-seq respectively. We then analyzed age-related changes in accessibility and how they relate to the transcriptome. Subsequently, we used an elastic net regression model to predict age from chromatin accessibility profiles with good accuracy. Finally, we characterized the clock by investigating its predictors and comparing its performance to that of transcriptome-based clocks.

## Results

### Profiling human blood samples over a wide age range

**Fig. 1:**
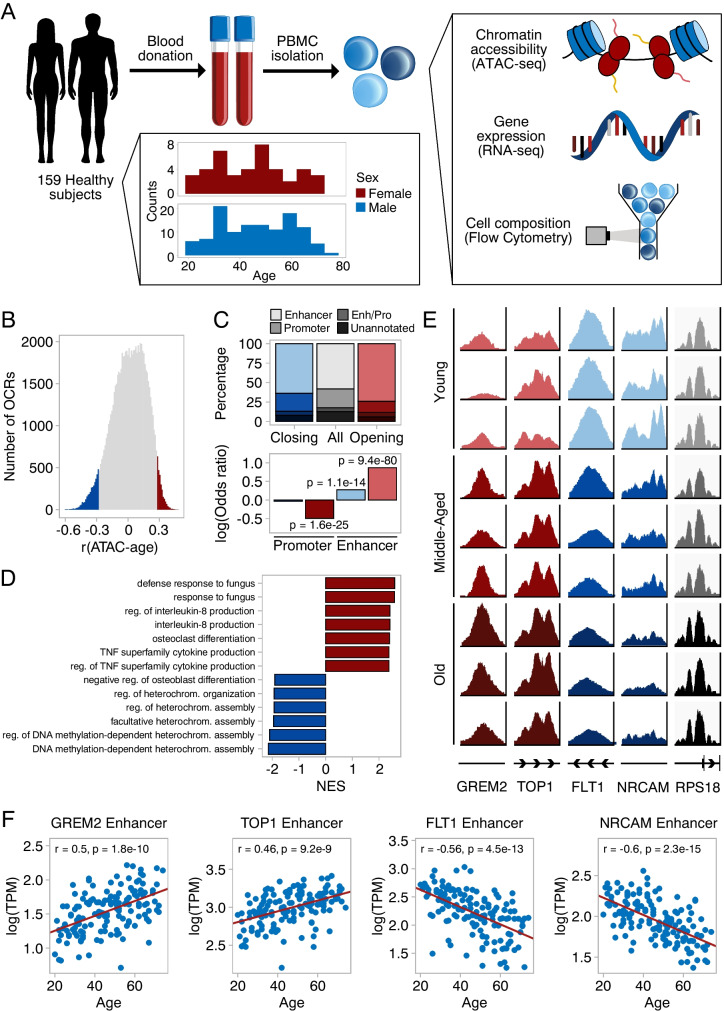
Chromatin accessibility changes during aging. **(A)** PBMCs were isolated from blood samples of 159 healthy donors with a broad age distribution (20—74). ATAC-seq, RNA-seq, and flow cytometry profiles were generated from all samples. **(B)** Distribution of correlations between chromatin accessibility and age (Spearman’s r). Statistically significant closing OCRs are highlighted in blue, while statistically significant opening OCRs are highlighted in red (FDR < 0.01). **(C)** Annotation of statistically significant OCRs to regulatory elements. Enrichment for promoters and enhancers among opening and closing OCRs. Log(odds ratios) and p-values were calculated using Fisher’s Exact test. **(D)** GSEA of chromatin accessibility changes during aging. Gene ontology biological process terms are plotted against the normalized enrichment score (NES). Terms with the top six positive (red) and negative (blue) NES are shown. **(E)** Accessibility profiles at the top two opening and closing OCRs, shown for representative samples of different ages. The y axis was rescaled using the same scale factors used for normalization of raw counts. The last column represents a housekeeping gene whose accessibility did not change during aging. Young: 20—22 years; Middle-aged: 45—47 years, Old: 70—71 years. **(F)** Scatterplots of chromatin accessibility (log(TPM)) against age of four OCRs with the strongest age correlation. Pearson’s r and p-values are indicated

Blood samples were acquired from 159 healthy donors (117 men, 42 women) covering an age range from 20 to 74 years (Fig. 1a). Peripheral blood mononuclear cells (PBMCs) were isolated to generate ATAC-seq profiles from 157 samples, of which 143 (105 men, 38 women) passed quality controls (age and sex distributions are included in Supplementary Fig. 1a. A representative histogram of fragment size distribution is included in Supplementary Fig. 2b). From these samples, we detected a total of 80,400 open chromatin regions (OCRs), of which 24.1% lay within 1 kbp of transcription start sites (TSS) and were thus annotated as promoters, 58.2% contained sites with reported enhancer activity, 5.0% were annotated as both promoters and enhancers. The remaining 12.7% OCRs which did not lie in the proximity of TSSs and had no reported enhancer activity will be referred to as “unannotated” (Fig. 1c). Principal component analysis of accessibility profiles placed samples on an aging trajectory along PC1 (PC1-age Pearson’s r = 0.35, p = 2.14e-5, Supplementary Fig. 1c). Additionally, we performed RNA-seq on all 159 samples, from which we detected the expression of 16,155 genes. Of these samples, 144 passed quality control (age and sex distributions are included in Supplementary Fig. 1b) and 132 had a matching ATAC-seq sample, which also passed quality control. Principal component analysis of expression profiles placed samples on an aging trajectory along PC1 and 2 (PC1-age Pearson’s r = -0.27, p = 9.89e-4, PC2-age Pearson’s r = 0.26, p = 1.86e-3, Supplementary Fig. 1c). Finally, we used flow cytometry to measure the proportions of monocytes, granulocytes, lymphocytes, total T cells, CD4 + T cells, CD8 + T cells, B cells, and NK cells in all samples (Supplementary Fig. 2a, Supplementary Fig. 3a—g). During aging, we detected an increase in the proportions of NK cells (Pearson’s r = 0.31, p = 1e-4) and a decrease in the numbers of total T cells (Pearson’s r = -0.22, p = 5.3e-3) and CD8 + T cells (Pearson’s r = -0.24, p = 2.4e-3). The proportions of monocytes, granulocytes, lymphocytes, CD4 + T cells, and B cells did not significantly correlate with age. Similar changes in PBMC compositions have been reported in previous studies [22, 28, 29].

### Chromatin accessibility changes in a site-specific manner during aging

To understand the effect of aging on the epigenome, we analyzed global and site-specific changes in chromatin accessibility. Based on the heterochromatin loss theory of aging we might expect to see a gradual de-repression of chromatin outside our OCRs. The fraction of reads within OCRs showed a negative trend during aging, but it was not significant (Pearson’s r = -0.13, p = 0.12, Supplementary Fig. 4a). We do however note that the fraction of reads within OCRs is sensitive to technical variation. Therefore, we asked if we could observe global changes through other means, such as changes in OCR width at promoters or de-repression of repetitive elements. The average coverage profile around transcription start sites (TSS) did not differ between young (< 35 years old, n = 40) and old donors (> 55 years old, n = 44) (Supplementary Fig. 4c). Similarly, we saw no significant increase in accessibility of repetitive elements in general (Pearson’s r = 0.13, p = 0.14, Supplementary Fig. 4b, d) nor when considering repetitive element families individually (Supplementary Fig. 4e).

As for site-specific changes, we observed a consistent opening of chromatin with age in 2622 OCRs, and closing in 3765 OCRs (Spearman’s r, FDR < 0.01, Fig. 1b). Several examples of coverage profiles for OCRs that open, close, or do not change with age are shown in Fig. 1e, whereas Fig. 1f shows the correlation between the accessibility of the same OCRs and age. Among the opening OCRs, 6.0% were annotated as both promoters and enhancers, 14.3% as promoters, 74.0% as enhancers, and 5.8% were unannotated. Among the closing OCRs, 5.4% were annotated as promoters and enhancers, 23.1% as promoters, 63.7% as enhancers, and 7.8% were unannotated. Interestingly, we observed significant enrichment of enhancers in both the opening and the closing OCRs compared to the background (Fisher’s Exact Test p = 9.44e-80 for opening, p = 1.08e-14 for closing, Fig. 1c). Conversely, promoters were depleted in the opening OCRs but not in the closing ones (Fisher’s Exact Test p = 1.6e-25 for opening, p = 0.38 for closing). This suggests that enhancers could be particularly sensitive to changes in accessibility during aging. Next, we linked OCRs to genes and investigated their involvement in biological processes. We associated OCRs with their closest gene and performed GSEA [30] (Fig. 1d, Supplementary File 4). Terms with a positive enrichment score included regulation of IL8 and TNF production and defense to fungus, whereas terms with a negative enrichment score were related to regulation of heterochromatin assembly, including heterochromatin assembly dependent on DNA methylation. We additionally performed GSEA on promoters only (Supplementary Fig. 5a) or associating enhancers to downstream genes using the PEREGRINE dataset: a collection of enhancer-gene links predicted based on ChIA-PET, eQTL, and Hi-C of multiple tissues, including blood [31] (Supplementary Fig. 5b). As with the previous method, terms with a positive enrichment score were associated with inflammation, while terms with a negative enrichment score were associated with regulation of chromatin assembly.

In conclusion, we found that chromatin accessibility of PBMCs does not undergo significant global changes during aging, at least in the age range we analyzed (20–74 years). Instead, we detect changes in specific regulatory elements, most commonly enhancers, which are associated with increased inflammation and reduced heterochromatin assembly. It is particularly puzzling to see age-related repression of OCRs upstream of genes involved in heterochromatin assembly, without observing significant global de-repression. It is also worth mentioning that ATAC-seq can only provide relative quantifications of accessibility, and therefore, a genome-wide, uniform increase in accessibility might be undetectable. Nonetheless, the correlations between accessibility at specific OCRs and age suggest that it should indeed be possible to construct an aging clock based on chromatin accessibility similar to what was done for DNA methylation.

### Age-related changes in chromatin accessibility relate to coherent changes in expression

One limitation of aging clocks based on DNA methylation is that changes in methylation are difficult to relate to downstream cellular processes, thus limiting their interpretability. A previous study found that transcription of genes downstream differentially methylated regions did not generally change in accordance with methylation during age [18]. The same study found that hypermethylation mostly affected genes whose expression was already low, thus explaining the apparent lack of effect on transcription.

**Fig. 2:**
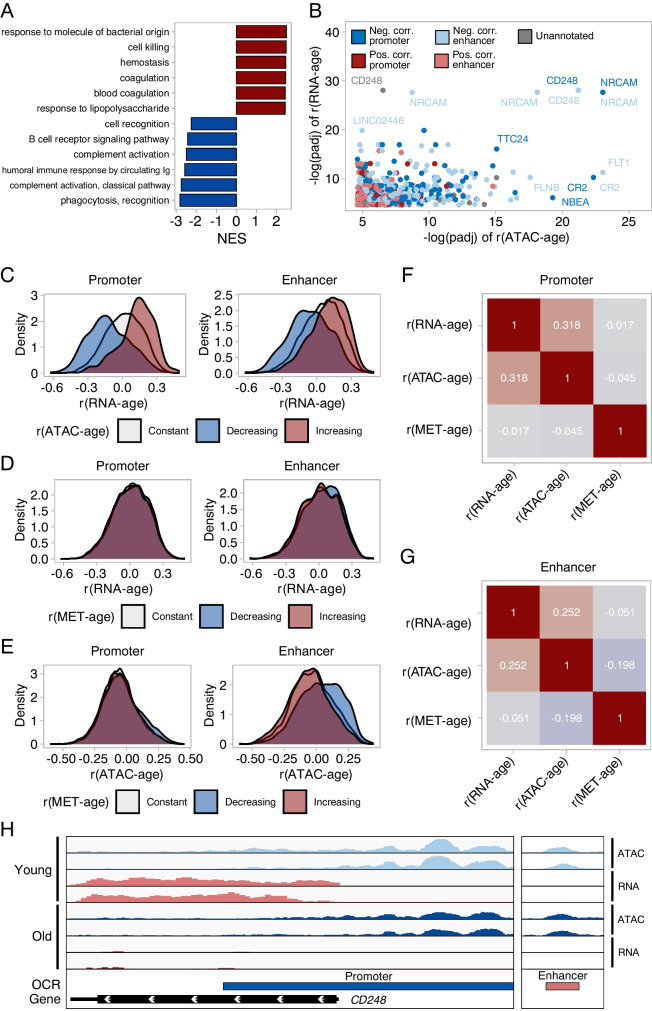
Integrative analysis of gene expression, chromatin accessibility and DNA methylation during aging. **(A)** GSEA of gene expression changes during aging. Gene ontology biological process terms are plotted against the normalized enrichment score (NES). Terms with the top six positive (red) and negative (blue) NES are shown. **(B)** Genes whose expression and accessibility at regulatory elements both correlated with age (Spearman’s r). The x-axis represents the significance of correlation in the ATAC-seq data while the y-axis represents the significance of correlation in the RNA-seq data. The significance of the correlation is represented by the -log of FDR-corrected p-values. **(C)** Distribution of gene expression age correlations of genes linked to promoters/enhancers which open during aging (Spearman’s r > 0, FDR < 0.01), close during aging (Spearman’s r < 0, FDR < 0.01) or do not change (FDR ≥ 0.01). **(D)** Distribution of gene expression age correlations of genes linked to CpGs in promoters/enhancers which gain methylation during aging (Spearman’s r > 0, FDR < 0.01), lose methylation during aging (Spearman’s r < 0, FDR < 0.01) or do not change (FDR ≥ 0.01). **(E)** Distribution of accessibility age correlations of OCRs containing CpGs which gain methylation during aging (Spearman’s r > 0, FDR < 0.01), lose methylation during aging (Spearman’s r < 0, FDR < 0.01) or do not change (FDR ≥ 0.01). **(F)** Pairwise correlations between gene expression age correlations, accessibility age correlations, CpG methylation age correlations, specifically in promoter regions. **(G)** Pairwise correlations between gene expression age correlations, accessibility age correlations, CpG methylation age correlations, specifically in enhancer regions. **(H)** ATAC-seq and RNA-seq coverage tracks for the gene CD248, whose expression and accessibility at promoter and enhancer both decrease with age. Two young and two old samples are shown. The y axis was rescaled using the same scale factors used for normalization of raw counts

Therefore, we asked if in our dataset we would be able to observe changes in expression coherent with the changes in chromatin accessibility. Out of 16,155 expressed genes, 440 were increasing in expression with age while 544 were decreasing (Spearman’s r, FDR < 0.01, Supplementary Fig. 5c). Terms with positive enrichment scores in GSEA (Fig. 2a, Supplementary File 7) related to pathogen response (response to molecule of bacterial origin, response to lipopolysaccharide) and coagulation (hemostasis, coagulation). Terms with negative enrichment scores related to B cell activity and complement activation (B cell receptor signaling pathway, complement activation, humoral response by circulating Ig). These terms suggest alterations in immune function and inflammation, coherently with what we saw for chromatin accessibility. Terms related to heterochromatin assembly were also enriched in our transcriptomic data but to a lower extent than for chromatin accessibility. Investigating in more detail, we identified several genes whose expression and accessibility at regulatory elements both correlated to age (Fig. 2b, h shows coverage plots of one such gene: CD248) and sought to determine if these were more common than expected by chance. Therefore, we compared the age correlations of genes linked to OCRs with a positive correlation with age (Spearman r > 0, FDR < 0.01), negative correlation with age (Spearman r < 0, FDR < 0.01), and no correlation with age (FDR > 0.01). We found that overall, genes linked to promoters whose accessibility increased with age were upregulated during aging (one-tailed Kolmogorov–Smirnov Test, D = 0.33, p < 0.001, Fig. 2c), similarly genes linked to promoters that closed with age tended to be downregulated in aging (one-tailed Kolmogorov–Smirnov Test, D = 0.34, p < 0.001, Fig. 2c). This pattern was weaker when we looked at enhancers whose accessibility increased with age (one-tailed Kolmogorov–Smirnov Test, D = 0.15, p < 0.001, Fig. 2c) and enhancers whose accessibility decreases with age (one-tailed Kolmogorov–Smirnov Test, D = 0.25, p < 0.001, Fig. 2c), but still highly significant. We repeated this analysis using the PEREGRINE gene-enhancer links but found that doing this reduced the agreement between chromatin accessibility and transcriptomic data (Supplementary Fig. 5d). The agreement did not improve even when only considering gene-enhancer links that were validated in blood (Supplementary Fig. 5d). Finally, we wondered how the strength of the relationship between accessibility and transcription compared to the association between methylation level and transcription. Thus, we used a publicly available DNA methylation dataset from Hannum et al. [6] to compute methylation-age correlations (Spearman’s r) and evaluated the pairwise correlations between age correlations of transcription, accessibility and methylation, genome-wide (Fig. 2f, g). We found that age-related changes in methylation had almost no correlation with transcriptomic alterations, both in enhancers and promoters (Pearson’s r = -0.017 and -0.051 respectively). In comparison, changes in accessibility correlated with changes in transcription, particularly at promoters (Pearson’s r = 0.318 for promoters, r = 0.252 for enhancers). Interestingly, changes in accessibility were correlated with methylation changes at enhancers (r = -0.198) but weakly at promoters (r = -0.045). We also repeated this comparison focusing on CpGs whose methylation level was significantly correlated with age (Spearman’s r, FDR < 0.01, Fig. 2d, e) to ensure that we would not miss non-linear relationships. Age correlations of genes downstream hypomethylating CpGs were significantly shifted towards positive values, albeit with minuscule effect sizes, at both promoters and enhancers (One-tailed Kolmogorov–Smirnov test, D = 0.022, p = 4.3e-6 for promoters, D = 0.055, p = 4.7e-11 for enhancers). The converse was true for genes downstream hypermethylating CpGs at promoters (D = 0.016, p = 2.8e-4) but not at enhancers (D = 0.020, p = 0.077 for enhancers). Thus, even when focusing on CpGs with extreme methylation changes during aging, we saw little effect on transcription.

It is however crucial to consider that in this comparison, the strength of association between methylation and transcription could be underestimated because the methylation and expression data was not produced in matched samples. To have a fairer comparison, we substituted our RNA-seq data with another PBMC RNA-seq dataset from Marquez et al. [22]. To our surprise, age-related changes in chromatin accessibility were still strongly correlated with transcriptomic alterations (Supplementary Fig. 5e, f, Pearson’s r = 0.314 for promoters, r = 0.235 for enhancers) while the correlation between methylation changes and expression changes remained weak (Pearson’s r = -0.023 for promoters, r = -0.046 for enhancers). Finally, prior reports have found the relationship between gene expression and gene body methylation can differ from the relationship between gene expression and promoter/enhancer methylation [32]. Thus, we correlated age-related changes in gene body methylation with age-related changes in gene expression but once again found very weak associations (Pearson’s r = -0.034 when using our RNA-seq data, r = -0.026 when using the Marquez et al. data. Methylation data from Hannum et al. was used in both cases).

Thus, we found that changes in chromatin accessibility during aging associate with coherent transcriptional alterations. Methylation changes, on the other hand, associated very weakly with age-related changes in expression. We therefore conclude that a clock constructed on chromatin accessibility would bear direct connection to transcriptomic alterations and their effect on biological processes.

### Chromatin accessibility predicts age and the effect of SARS-CoV-2 infection

**Fig. 3:**
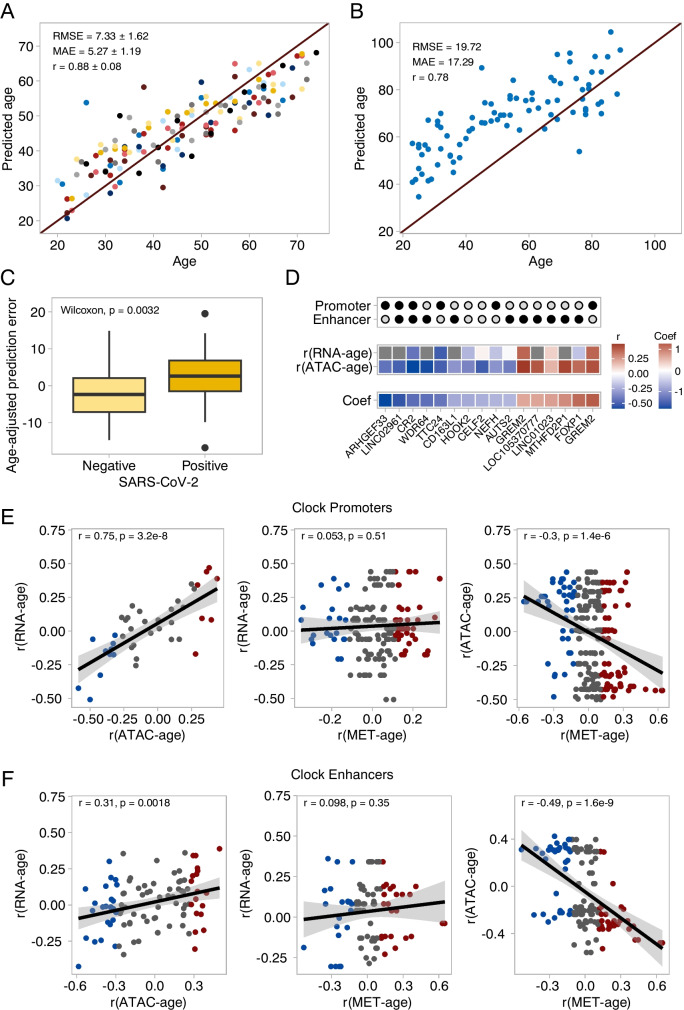
Chromatin accessibility predicts age. **(A)** Age predictions of the chromatin accessibility clock. The scatter plot shows the test set predictions from each outer fold of nested cross-validation (11 different models, each sample in the test set once). Mean and standard deviation for root mean squared error (RMSE), median absolute error (MAE), and Pearson correlation coefficient (r) are shown. **(B)** Age predictions of a clock trained on all our chromatin accessibility data and tested on an external dataset by Marquez et al. (22) RMSE, MAE, and Pearson’s r are indicated. **(C)** Age predictions on SARS-CoV-2 positive and negative patients. Prediction errors were adjusted to account for overestimation of age of young individuals compared to old. Statistical tests for unadjusted predictions are included in the main text. **(D)** Annotation of the 16 OCRs with the higher absolute coefficients in the final model ranked. Clock coefficients and age correlation of chromatin accessibility and gene expression level are shown for each OCR/gene pair. **(E)** Relationship between changes in gene expression, chromatin accessibility, and DNA methylation at promoters selected by the clock. **(F)** Relationship between changes in gene expression, chromatin accessibility, and DNA methylation at enhancers selected by the clock

Having found many site-specific changes in chromatin accessibility with age, we investigated whether these changes could be used to predict the age of the blood donors. To do so, we trained an elastic net regression model on the 143 ATAC-seq samples which passed quality control. We used nested cross-validation to tune hyperparameters and estimate the performance of the model. Across the outer folds of the nested cross-validation, the model selected 183 ± 58 OCRs as predictors and predicted age with an RMSE of 7.33 ± 1.62, MAE of 5.27 ± 1.19, and r of 0.88 ± 0.08 (Fig. 3a). We then trained a final model on all our ATAC-seq samples and tested its performance on a completely distinct dataset by Marquez et al. [22] comprising 84 samples after quality control (Fig. 3b). The predictions provided by our model were highly correlated with the real ages of individuals (r = 0.78). However, the age of most individuals was overestimated, leading to large RMSE (19.72) and MAE (17.29). The reasons for this inaccuracy might be the usage of a different ATAC-seq protocol by Marquez et al. (The original ATAC-seq protocol [27] as opposed to Omni-ATAC [33]) and the different genetic backgrounds of the sample populations. We believe that expanding the training dataset to include samples generated in different manners would improve the clock’s resilience to batch effects. We additionally asked if our clock would be able to detect the effect of health conditions. A previous study by Giroux et al. collected ATAC-seq data from PBMCs of SARS-CoV-2 patients and healthy controls [34]. We trained an additional clock on samples from Marquez et al. and this study and compared the discrepancy between predicted age and real age for SARS-CoV-2 negative and positive individuals. We found that this discrepancy was higher in positive patients (T-test, p = 0.006). We noted that in both Fig. 3a and b, the clock tended to overestimate the ages of young individuals. This could confound the comparison of positive and negative patients. Thus, we repeated the comparison on SARS-CoV-2 positive and negative individuals while accounting for the effect of chronological age on clock accuracy using a linear model: the effect of infection remained significant and added 5 years to the predicted age of patients (SARS-CoV-2 + effect = 5.35, p = 0.005, Fig. 3c).

### The role of OCRs selected by the ATAC-clock

Next, we investigated the nature of the features of the final model: a total of 228 OCRs were selected, 116 of which were taken with a positive coefficient and 112 with a negative one. Of all the OCRs selected by the model, 7.5% were annotated as promoters and enhancers, 19.3% as promoters, 57.0% as enhancers, and 16.2% were unannotated. Interestingly, clock sites did not show enrichment for enhancers (Fisher’s exact test, odds ratio = 1.06, p = 0.73). This contrasts with the enhancer enrichment we saw in the set of age-correlated OCRs. A likely explanation for this is that elastic net models do not simply select features based on their correlation to the response variable but aim to eliminate redundant features. We then analyzed the relationship between the accessibility of OCRs selected by the clock and gene expression. As expected from our genome-wide analysis, we found a strong correlation between the age-correlation of accessibility at OCRs and the age-correlation of transcription at the respective downstream genes, both in promoters and enhancers (Fig. 3e, f). This signifies that the chromatin accessibility features selected by the clock can be directly related to transcriptomic changes and the biological processes associated with them.

With this knowledge, we investigated the clock OCRs with the largest absolute coefficients (Fig. 3d). Among these OCRs were both the promoter and enhancer of *GREM2,* a gene that encodes a senescence-associated secretory phenotype (SASP) factor with a known association with aging in adipose tissue and skin [35, 36]. The promoter of *GREM2* was also selected in every nested cross-validation model, highlighting its robustness to predict age. In our data, both the *GREM2* promoter (Spearman’s r = 0.44, q = 1.23e-5) and the GREM2 enhancer (Spearman’s r = 0.5, q = 3.83e-7) open with age and associated with increased transcription (Spearman’s r = 0.39, q = 2.1e-4). *CR2*, the gene that encodes the complement receptor type 2, has been previously shown to decline with age in B-cells and is associated with ischemic stroke, autoimmune disease, and chronic infection [37, 38]. In our data, chromatin accessibility at the *CR2* promoter/enhancer strongly decreased with age (Spearman’s r = -0.59, q = 2e-10), in agreement with CR2 expression (Spearman’s r = -0.42, q = 3.52e-5).

### The ATAC clock shares some links with methylation clocks

Next, we looked for similarities between our clock and previously published methylation-based aging clocks. The Hannum clock [6] was trained on whole blood and bases its predictions on 71 CpGs. Our clock includes two OCRs which span Hannum clock sites: the promoter of *ARHGEF33,* and an enhancer of *KLF13.* Interestingly, the promoter of *ARHGEF33* was the feature with the strongest coefficient in our clock (-1.79). The Horvath pan-tissue clock [7] bases its predictions on 353 CpGs, but none of these lied in the OCRs chosen by our clock, perhaps because the Horvath clock is trained on multiple tissues rather than just blood. Although few of our accessibility clock’s sites contained CpG sites used by the Hannum and Horvath clocks, we investigated if the age-related changes in accessibility observed in our data could be ascribed to changes in DNA methylation. We focused on the Hannum dataset as this was also obtained from blood samples. Of the 485,577 CpG markers in the Illumina Infinium 450 Human Methylation array, 168,778 fell within our OCRs (123,266 in promoters, 22,278 in enhancers, 14,501 in OCRs annotated as both promoters and enhancers, and 3859 in unannotated OCRs). We found that among enhancers selected by our clock, increased accessibility during aging correlated with decreased methylation and vice versa (Pearson’s r = -0.49, p = 1.7e-9, Fig. 3f). This pattern was also seen in promoters chosen by our clock, but to a lesser extent (Pearson’s r = -0.30, p = 1.4e-6, Fig. 3e). Despite the association between changes in accessibility and both transcription and methylation, changes in methylation did not directly relate to changes in transcription neither at promoters (Pearson’s r = 0.053, p = 0.51, Fig. 3e) nor at enhancers (Pearson’s r = 0.098, p = 0.35, Fig. 3f).

Thus, although our clock shares few sites with the Hannum clock and none with the Horvath clock, it appears that age-related changes in accessibility might be partially related to methylation changes. This is not surprising, considering that DNA methylation is one of the epigenetic mechanisms contributing to chromatin repression, which is in turn reflected in chromatin accessibility. Nonetheless, we consider it unlikely that the changes in chromatin accessibility used by our clock to predict age depend entirely on DNA methylation. Instead, the fact that changes in accessibility correlated with changes in gene expression, but changes in methylation did not, suggests that most age-related accessibility alteration could be the result of two overlapping processes: one with a direct effect on transcription (perhaps chromatin remodeling) and DNA methylation.

### Changes in chromatin accessibility predict age better than changes in gene expression

**Fig. 4:**
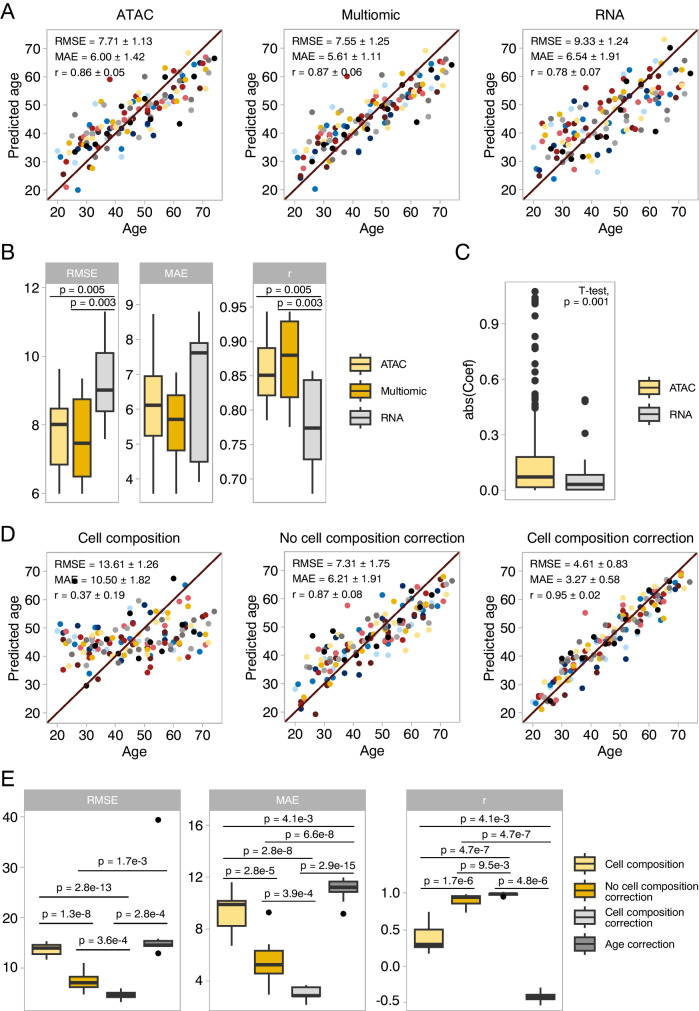
Chromatin accessibility allows for better age prediction than gene expression. Correcting for cell composition improves clock accuracy. **(A)** Age predictions of chromatin accessibility, transcriptomic and multiomic clocks trained on matched samples (n = 132). RMSE, MAE, and Pearson’s r are indicated. **(B)** Score comparison of the chromatin accessibility, transcriptomic and multiomic clocks. Inner boxplots depict medians and first and third quartiles, with whiskers extending up to 1.5 × interquartile range. p-values were calculated using a two-tailed T-test. **(C)** Absolute coefficients of gene and OCR features selected by the multiomic clock. Features were standardized prior to clock training, bringing gene expression and chromatin accessibility features to the same scale **(D)** Age predictions of clocks trained on cell composition alone, chromatin accessibility without cell composition correction, and chromatin accessibility with cell composition correction (n = 142). **(E)** Score comparison of the cell composition, chromatin accessibility, and corrected chromatin accessibility clocks

We wanted to compare the predictive power of our aging clock based on chromatin accessibility with that of clocks based on gene expression. Therefore, we used samples from donors for which we obtained both ATAC-seq and RNA-seq profiles to construct two separate clocks (Fig. 4a). In this direct comparison, the chromatin accessibility clock performed significantly better by two metrics (RMSE = 7.71 ± 1.13, MAE = 6.00 ± 1.42, and r 0.86 ± 0.05 for the chromatin accessibility clock compared with RMSE = 9.33 ± 1.24, MAE = 6.54 ± 1.91, and r = 0.78 ± 0.07 for the gene expression clock, two-tailed t-Test: p-values = 0.005 (RMSE), 0.46 (MAE), 0.005 (r), Fig. 4b). We then trained a third “multiomic” clock using concatenated chromatin accessibility and gene expression data (80,400 OCRs + 16,155 genes, Fig. 4a). This multiomic clock predicted age better than the transcriptomic clock but with similar accuracy to the chromatin accessibility clock (RMSE = 7.55 ± 1.4, MAE = 5.61 ± 1.64, and r = 0.87 ± 0.06, Fig. 4b). Moreover, a final multiomic clock trained on all samples showed some preference towards chromatin accessibility features rather than transcriptomic ones, albeit non-significantly (Selected OCRs: 281, selected genes: 41, Fisher’s exact test: odds ratio = 1.38, p = 0.06). Additionally, OCRs chosen by the multiomic clock had larger coefficients than the genes selected by the clock (Fig. 4c).

Thus, in our dataset, accessibility features appear to allow for better age predictions than gene expression features. However, we note that the BiT age clock was able to obtain better performance from gene expression data by binarizing the features in a dataset with a similar number of samples to ours: n = 131, RMSE = 8.41, MAE = 5.24, r = 0.96 [17]. Finally, using both chromatin accessibility and transcriptomic data to predict age does not significantly improve performance compared to chromatin accessibility alone, but might aid interpretation of the clock.

### Sex differences have little influence on the clock’s performance

Previous publications have reported important differences in immune aging between women and men [22]. We observed that our chromatin accessibility clock tended to underestimate the age of women compared to men (T-test: p = 0.051, Supplementary Fig. 6a). Thus, we wondered if correcting for sex would improve clock performance. A clock trained on sex-corrected data did not perform better than its uncorrected counterpart (Supplementary Fig. 6b, c). Therefore, it seems that sex differences do not have a large effect on clock performance.

### The clock relies on cell-intrinsic changes in chromatin accessibility rather than changes in cell composition

Finally, because our flow cytometry data revealed a correlation between the size of certain immune cell populations and age, we wanted to understand to what extent the performance of the ATAC clock depended on changes in cell composition as opposed to cell-intrinsic changes in accessibility. To this end, we trained clocks using 142 samples for which we had both chromatin accessibility and cell composition data (Fig. 4d). A clock trained solely on cell composition had terrible performance (RMSE = 13.61 ± 1.26, MAE = 10.50 ± 1.82, r = 0.37 ± 0.19). Additionally, when we trained a clock on both cell composition and chromatin accessibility features, none of the 11 models selected by nested cross-validation used cell composition features as predictors. However, this does not preclude that accessibility features could carry information on cell composition. Thus, we investigated how correcting chromatin accessibility for cell composition changes affected clock performance. A clock trained on cell composition corrected data was significantly more accurate (RMSE = 4.61 ± 0.83, MAE = 3.27 ± 0.58, r = 0.95 ± 0.02, Fig. 4e) than a clock train on the same uncorrected data (RMSE = 7.31 ± 1.75, MAE = 6.21 ± 1.91, r = 0.87 ± 0.08). Conversely, when we corrected for changes in chromatin accessibility that could not be explained by changes in cell compositions, we again obtained a clock with terrible performance (RMSE = 16.89 ± 7.51, MAE = 12.59 ± 0.96, r = -0.16 ± 0.22). Thus, cell composition variability seems to affect clock performance negatively, even though certain cell population sizes correlate with age. Although correcting for cell composition yielded impressive performance, we note that this is not a realistic scenario for usage of the clock: cell composition correction requires knowing the age of samples to separate cell intrinsic and extrinsic effects. To see if cell composition correction would be viable in absence of age information, we tried correcting for cell composition without preserving age effects, but this yielded a very imprecise clock (Supplementary Fig. 6d). Alternatively, we tried estimating correction coefficients on the training set and using them to apply the correction on the test set within nested cross-validation, but clock performance did not significantly improve compared to a clock trained on uncorrected data (Supplementary Fig. 6d).

Thus, despite the mild correlation between the size of certain cell populations and age, cell composition alone is not sufficient to predict age accurately. On the contrary, it seems that variation in cell composition decreases accuracy of the clock by introducing noise to chromatin accessibility data. This noise could reflect environmental effects such as recent exposure to pathogens, which could partially mask age-related changes in cell composition. Unfortunately, correction for cell composition in absence of age information did not improve clock performance compared to uncorrected data. Nonetheless, a larger training dataset could allow for a better estimation of correction coefficients. In that case, the performance gain would need to be substantial to justify collecting flow cytometry data on top of performing ATAC-seq.

## Discussion

One major limitation of epigenetic clocks lies in their difficult interpretability [39]. In particular, age-related changes in CpG methylation have been reported to correlate poorly with transcription of downstream genes, making it difficult to draw a link between altered methylation and disruptions in cellular function [18]. To solve this, we have investigated the suitability of chromatin accessibility as a new biomarker of aging.

Initially, we analyzed the effect of aging on global and local chromatin accessibility. We found that age-related changes were mostly focal, and preferentially affected enhancers. The lack of significant global changes in accessibility contrasts with the heterochromatin loss theory of aging and with several observations of loss of repressive histone marks such as H3K9me3 and H3K27me3 [24, 25]. It is important to note that ATAC-seq only allows for relative quantifications of accessibility, thus a global, uniform increase in chromatin accessibility would not have been detected with our methods. Nonetheless, we saw no significant gain in accessibility at repetitive elements, change in TSS accessibility profiles, or flattening of the epigenetic landscape as would be indicated by a reduction in FRIP. Thus, the term “heterochromatin redistribution” may be more appropriate to describe the changes that the epigenome undergoes during aging. As to why enhancers appeared more prone to change accessibility during aging, we hypothesize that accessibility at enhancers may be more dynamic and thus more affected by local or systemic changes in the cellular environment. Future studies might investigate which chromatin factors (histone variants, post-translational modifications, transcription factors etc.) drive the observed changes in accessibility specifically at enhancers.

Next, we investigated the link between gene transcription and chromatin accessibility at corresponding regulatory elements. In general, age-related changes in transcription and accessibility were related to similar biological processes, and at a site-specific level, we found that accessibility changes at promoters and enhancers associated with coherent transcriptional responses during aging. In contrast, DNA methylation exerted a weak effect on gene expression, as expected based on previous findings [18]. It follows that a chromatin accessibility clock would have a clearer link to cellular function than DNA methylation clocks, thus providing better interpretability.

We then showed for the first time that chromatin accessibility profiles of PBMCs can be used to predict the age of donors, with an RMSE of 7.33 years, MAE of 5.27 years, and r of 0.88. Importantly, we trained and validated our clock using nested cross-validation, meaning that the test sets used to evaluate clock performance were not included in the training and hyperparameter tuning process, leading to unbiased performance estimation. The clock predicts age accurately, although we expect performance could be improved further by adding more samples to the training set, as state-of-the-art methylation clocks have typically been trained on thousands of samples. When we tested our clock on previously published data generated with a different ATAC-seq protocol, we found that age predictions were highly correlated with chronological ages but tended to overestimate the actual value. We recommend the use of the Omni-ATAC protocol to any researcher interested in using our clock, or perform ATAC-seq in general, as Omni-ATAC provides higher quality data, in part by reducing mitochondrial DNA contamination [33]. Unfortunately, we were limited in our work by the scarce availability of ATAC-seq data with reported age information. We believe that as more data becomes available, ATAC clocks could be trained to better tolerate differences in protocols and batch effects. We tested our clock on an additional public dataset, comprising ATAC-seq data of SARS-CoV-2 positive and negative individuals, finding that the infection associated with higher age predictions. A recent study has found a similar, transient increase in predicted age using methylation clocks [40]. Without matched cell composition data, we are not able to conclude whether this effect is mainly cell-intrinsic or driven by changes in circulating cell populations during infection. Nonetheless, this could signify that the systemic inflammation caused by SARS-CoV-2 infection bears resemblance to inflammaging: chronic age-related increase in levels of inflammatory markers, which comprises both cell intrinsic and compositional immune dysregulation [29, 41].

Since we had matched ATAC-seq and RNA-seq profiles, we could directly compare the performance of our chromatin accessibility clock to a transcriptomic counterpart. In this direct comparison, our ATAC-clock performed significantly better. When we additionally developed a multiomic clock based on chromatin accessibility and transcriptome features, we saw that the multiomic clock performed similarly to the accessibility clock and relied on accessibility features more than on gene expression features, once again suggesting that chromatin accessibility data may allow for better age prediction than transcriptomic data. It is possible that gene expression varies more rapidly than chromatin accessibility (for example, in response to stress, circadian regulation etc.) thus introducing more noise to the prediction.

Finally, we investigated the relative importance of changes in cell composition as opposed to cell-intrinsic changes in accessibility and found that cell composition correction improved the performance of the clock. Cell composition may depend on recent exposure to pathogens, explaining why correction for cell composition may be beneficial.

## Conclusion

We have shown the feasibility of an epigenetic clock based on chromatin accessibility, which bears a strong relationship with transcription while performing better than transcriptomic clocks. We hope that this will provide the field with a new method to produce interpretable aging clocks.

## Methods

### Blood collection

Anonymized whole blood from 159 donors between the ages of 20 and 74 was obtained from the Interregional Blood Transfusion center in Lausanne-Epalinges, Switzerland. The internal review board approved the study, and all donors gave written consent to the use of their blood for research purposes. Samples were processed within 4.5 h after blood collection.

### PBMC isolation

Blood was diluted with equal amounts of Dulbecco’s phosphate-buffered saline (Gibco) and layered on top of Histopaque-1077 (Sigma-Aldrich). Density gradient centrifugation was carried out according to the manufacturer’s protocol and PBMCs were collected and washed. Cells were counted on a LUNA-II Automated Cell Counter (Logos Biosystems) and immediately aliquoted for ATAC-Seq library preparation, RNA extraction, and PBMC staining/fixation. All protocols were carried out simultaneously.

### ATAC-Seq library preparation

ATAC-Seq library preparation was performed according to the Omni-ATAC protocol [33] using Tn5 provided by the EPFL Protein Production and Structure facility. Transposed fragments were purified using the MinElute PCR Purification Kit (Qiagen). The eluate was PCR amplified using 2 × NEBNext Master Mix (NEB) and pre-mixed primers with unique dual indexes for Illumina sequencing (IDT). The library was purified by double-sided bead size selection using SPRIselect (Beckman Coulter).

### RNA extraction

RNA extraction was performed using the Monarch Total RNA Miniprep Kit (NEB) according to the manufacturer’s protocol.

### PBMC staining and flow cytometry

Cells were stained with Ghost-Dye/V510, CD3 + /V421, CD4 + /FITC, CD8 + /APC-Cy7, CD16 + /PE, CD19 + /PE-Cy7 and CD56 + /APC (Biolegend). Cells were fixed in Fixation and Permeabilization Solution (BD). A Cytoflex S flow cytometer (Beckman Coulter) was used to analyze the subpopulation ratios.

### ATAC sequencing and pre-processing

ATAC-Seq libraries were subjected to 150 bp paired-end sequencing on an Illumina NovaSeq 6000 by Novogene (UK) Company Limited with a sequencing depth of 30 million reads. Raw reads were adapter and quality trimmed using Trim Galore! [42] and mapped to the GRCh38 build of the human genome using bowtie2 (with settings –very-sensitive -X 1000 –dovetail) [43]. Before peak calling, raw bams were filtered to remove reads with multiple mappings, PCR duplicates, and mitochondrial reads using samtools [44] and Picard tools [45].

Alignments in BAM format were converted to BED and used to call peaks with MACS2 (with settings -f BED -g "hs" –keep-dup "all" -q 0.01 –nomodel –shift -100 –extsize 200) [46]. To define a common peakset, we initially computed the union of all individual peak sets using BEDTools merge on the narrowPeak MACS2 outputs [47]. Next, we identified regions which were reliably called as peaks across multiple samples using BEDTools multiinter and filtering the output to regions called in 50 samples or more. We then filtered the union peakset to only peaks containing at least one of the reliably called regions using BEDTools intersect. Finally, we discarded peaks overlapping the ENCODE blacklist [48].

We generated a raw count table using featureCounts [49], specifically counting Tn5 cut sites rather than whole fragments. Counts were first transformed to read densities by dividing the counts at each peak by the length of the peak in kilobases and then normalized by dividing by the total number of reads-in-peaks in millions.

We considered samples with less than 11 million good quality alignments and/or FRIP below 0.18 as low quality. We additionally discarded outliers in our samples using the elliptic envelope method on the first two principal components of the normalized counts in log scale. This removed 16 samples, including all samples with low sequencing depth and low FRIP.

Samples in the Marquez et al. [22] and Giroux et al. [34] datases used for testing the ATAC clock were processed starting from raw reads and subsequently mapped and filtered as our own samples. Peak calling, however, was not performed, and instead, the reads were counted over the peakset generated on our data alone. Outliers were removed as with our own dataset, but we did not impose a minimum FRIP, since we did not perform peak calling on these samples.

Coverage bigWig tracks were generated from regions centered around Tn5 cut sites using deepTools bamCoverage [50] with the same scaling factors used to normalize the counts. TSS profiles were generated from the bigwig tracks using deeptools computeMatrix and plotProfile.

### RNA sequencing and data pre-processing

RNA-Seq library preparation and sequencing were performed by Novogene (UK) Company Limited on an Illumina NovaSeq 6000 in 150 bp paired-end mode. Raw FASTQ files were assessed for quality, adapter content, and duplication rates with FastQC. Reads were aligned to the Human genome (GRCh38) using the STAR aligner (v2.7.9a) [51] with '–sjdbOverhang 100'. The number of reads per gene was quantified using the featureCounts function in the subread package [49]. Ensembl transcripts were mapped to gene symbols using the mapIds function in the AnnotationDbi package [52] with the org.Hs.eg.db package [53]. EdgeR was used to normalize row counts using the trimmed means of M-values method and filter low expression genes [54]. Finally, 15 outliers were removed using the same strategy employed for the ATAC-seq dataset. We used the same pipeline to process the RNA-seq data by Marquez et al.

### Clock construction and characterization

Training and validation of the elastic net model were carried out in Python using the Scikit-learn module [55]. Features were standardized prior to training using a StandardScaler. Samples were assigned to 11 groups so that the age composition in each group would cover the age range uniformly. Nested cross-validation was used to tune hyperparameters and estimate the performance of the model. Both the outer and inner cross-validation loops were run as leave-one-group-out cross-validation, meaning that the outer loop used each of the 11 groups once as a test set, while the inner loop alternated over the remaining 10 groups. The performance of the models is reported using root mean squared error (RMSE), median absolute error (MAE), and the Pearson correlation coefficient (r). Whenever multiple clocks were compared against each other, they were trained on samples sourced from the same donors and using the same partitions for cross-validation. Correction for sex and cell composition was performed in R using removeBatchEffect in the limma package [56], after preparing the data with voom [57]. Unless otherwise specified, age information was included into the experimental design to preserve the age effect. When correction for cell composition was included as part of clock training, we fitted models explaining accessibility of each OCRs in the training set as a function of age and cell composition using a MultiOutputRegressor with LinearRegression. The fitted coefficients for cell composition features were then used to subtract the effect of cell composition from train and test sets.

### Annotation of OCRs and repetitive elements

OCRs were annotated as promoters if they lay 1000 bp upstream or downstream of the transcription start sites. OCRs were annotated as enhancers if they overlapped regions annotated as enhancers in the PEREGRINE dataset [31]. Notably, we allowed OCRs to be annotated both as promoters and enhancers. In the case of promoters, OCRs were linked to the closest gene, whereas for enhancers, we tested linking to the closest gene or using the gene-enhancer links in the PEREGRINE dataset. Repetitive regions of the genome were identified using repeat masker [58].

### Statistical analysis

Statistical analysis was carried out in R. GSEA was performed using the ClusterProfiler R package with 1000 permutations [59]. When performing GSEA on ATAC-seq data, we created a ranked list of genes with the following procedure: 1) Starting from genes-OCR links generated as described above we discarded OCRs with no linked gene 2) We discarded OCR-gene pairs in which the gene was not expressed. 3) When a gene was linked to multiple OCRs we selected the OCR-gene pair for which chromatin accessibility and expression were best correlated across the set of samples common to our ATAC-seq and RNA-seq data. This produces 1:1 OCR-gene links. Alternatively, we selected promoter-gene pairs only, which also yields 1:1 links 4) We ranked genes based on the spearman correlation between chromatin accessibility at their linked OCR and age.

### Supplementary Information

Below is the link to the electronic supplementary material.

Supplementary file1 (PDF 39 KB)

Supplementary file2 (PDF 91 KB)

Supplementary file3 (PDF 364 KB)

Supplementary file4 (PDF 94 KB)

Supplementary file5 (PDF 37 KB)

Supplementary file6 (PDF 38 KB)

Supplementary file7 (TSV 35 KB)

Supplementary file8 (TSV 208164 KB)

Supplementary file9 (TSV 9465 KB)

Supplementary file10 (CSV 309 KB)

Supplementary file11 (TSV 42846 KB)

Supplementary file12 (TSV 1312 KB)

Supplementary file13 (CSV 598 KB)

Supplementary file14 (CSV 42 KB)

Supplementary file15 (TSV 15 KB)

## Data Availability

All raw and processed data generated in this study are available on GEO (GSE193142). We additionally used publicly available methylation data by Hannum et al. [6] (GSE40279), SARS-CoV-2 patient ATAC-seq data by Giroux et al. [34] (GSE206284). The ATAC-seq and RNA-seq data generated by Marquez et al. [22] which we used for validation is under controlled access of the original authors. The code used to process raw sequence data, train the clocks and generate figures is available at https://github.com/SunScript0/ATAC-clock
