# Supplementary figures and images for: ATAC-clock: An aging clock based on chromatin accessibility
Source: GeroScience. 2023 Nov 4;46(2):1789–806. doi: 10.1007/s11357-023-00986-0 (PMC10828344; doi:10.1007/s11357-023-00986-0)

Supplementary Figure 1

A

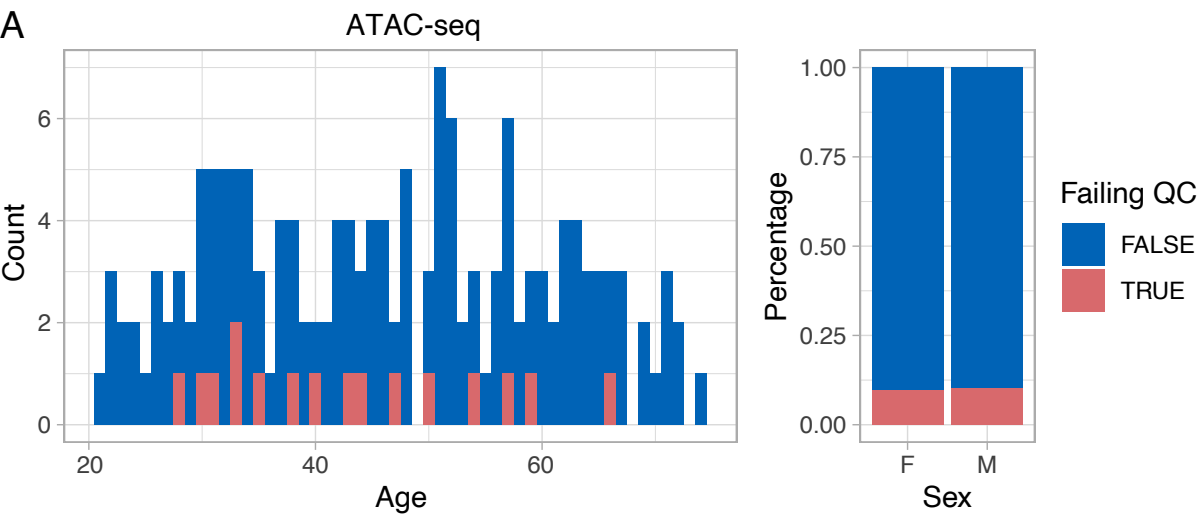

B

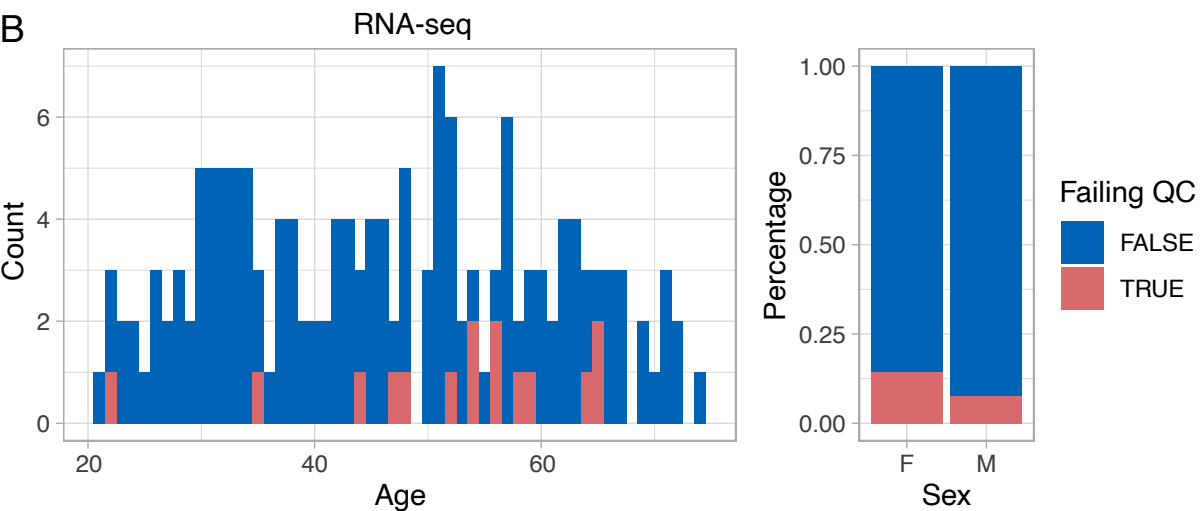

C

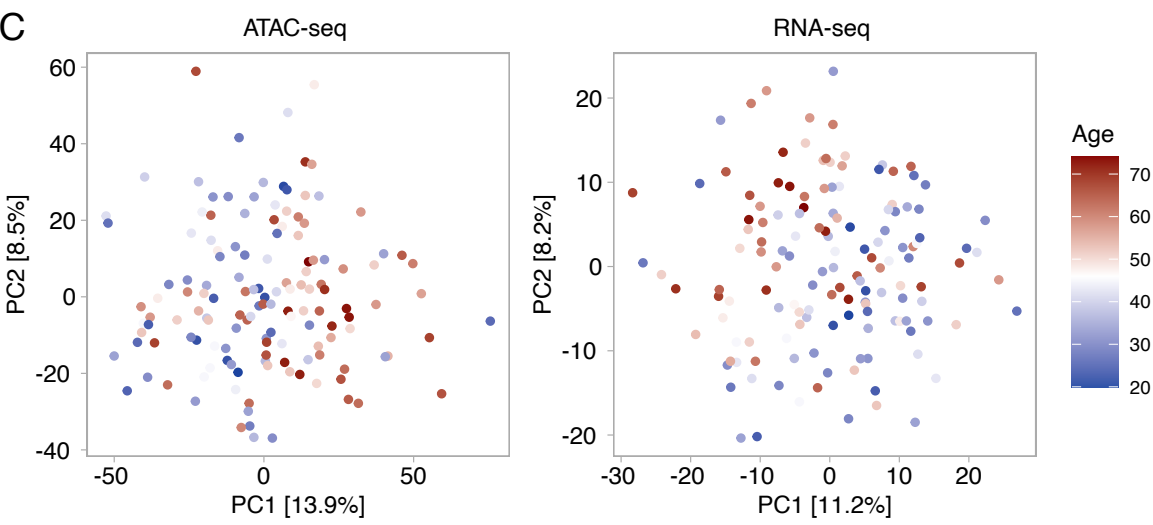

Supplement: Supplementary file 1 — Supplementary file1 (PDF 39 KB) [file 11357_2023_986_MOESM1_ESM.pdf]

Supplementary Figure 2

A

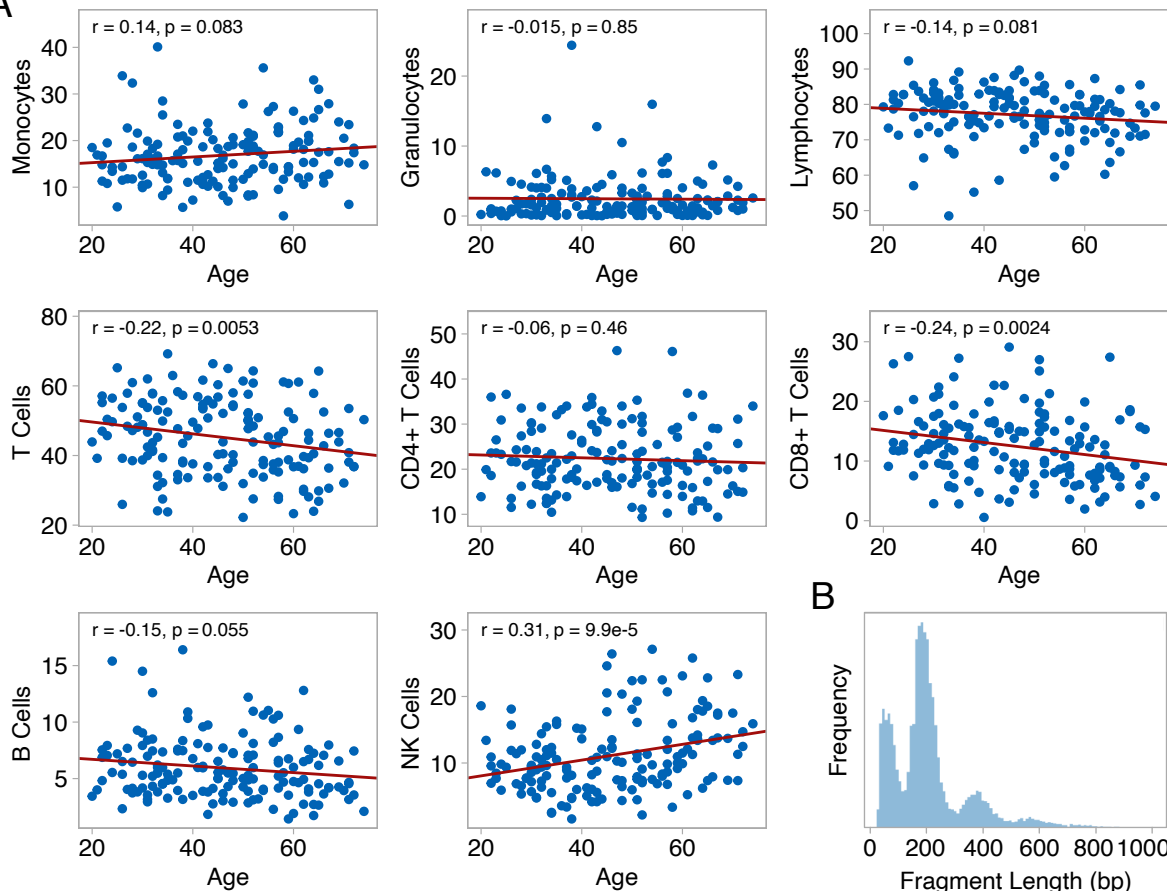

Supplement: Supplementary file 2 — Supplementary file2 (PDF 91 KB) [file 11357_2023_986_MOESM2_ESM.pdf]

Supplementary Figure 3

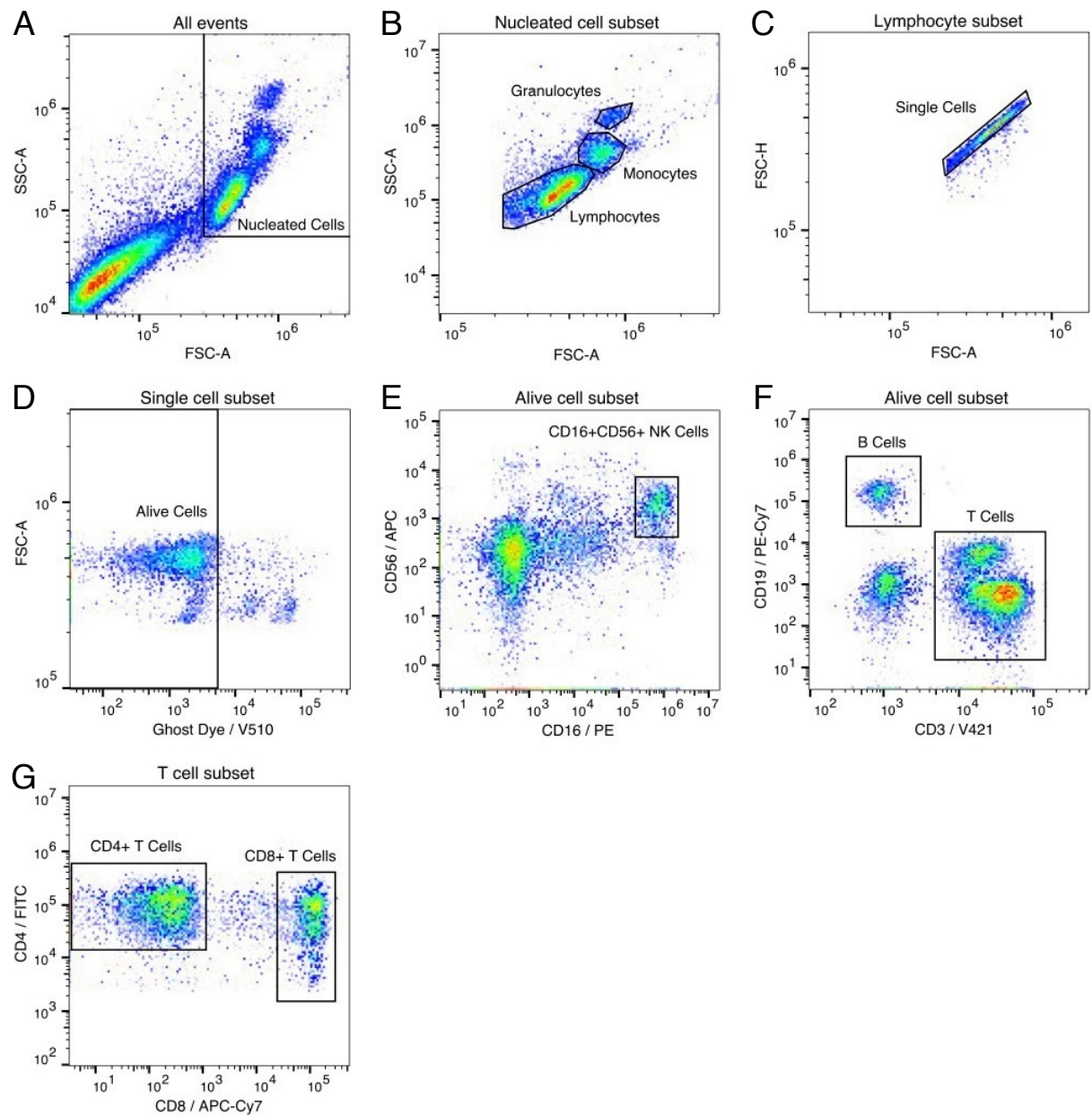

Supplement: Supplementary file 3 — Supplementary file3 (PDF 364 KB) [file 11357_2023_986_MOESM3_ESM.pdf]

Supplemental Figure 4

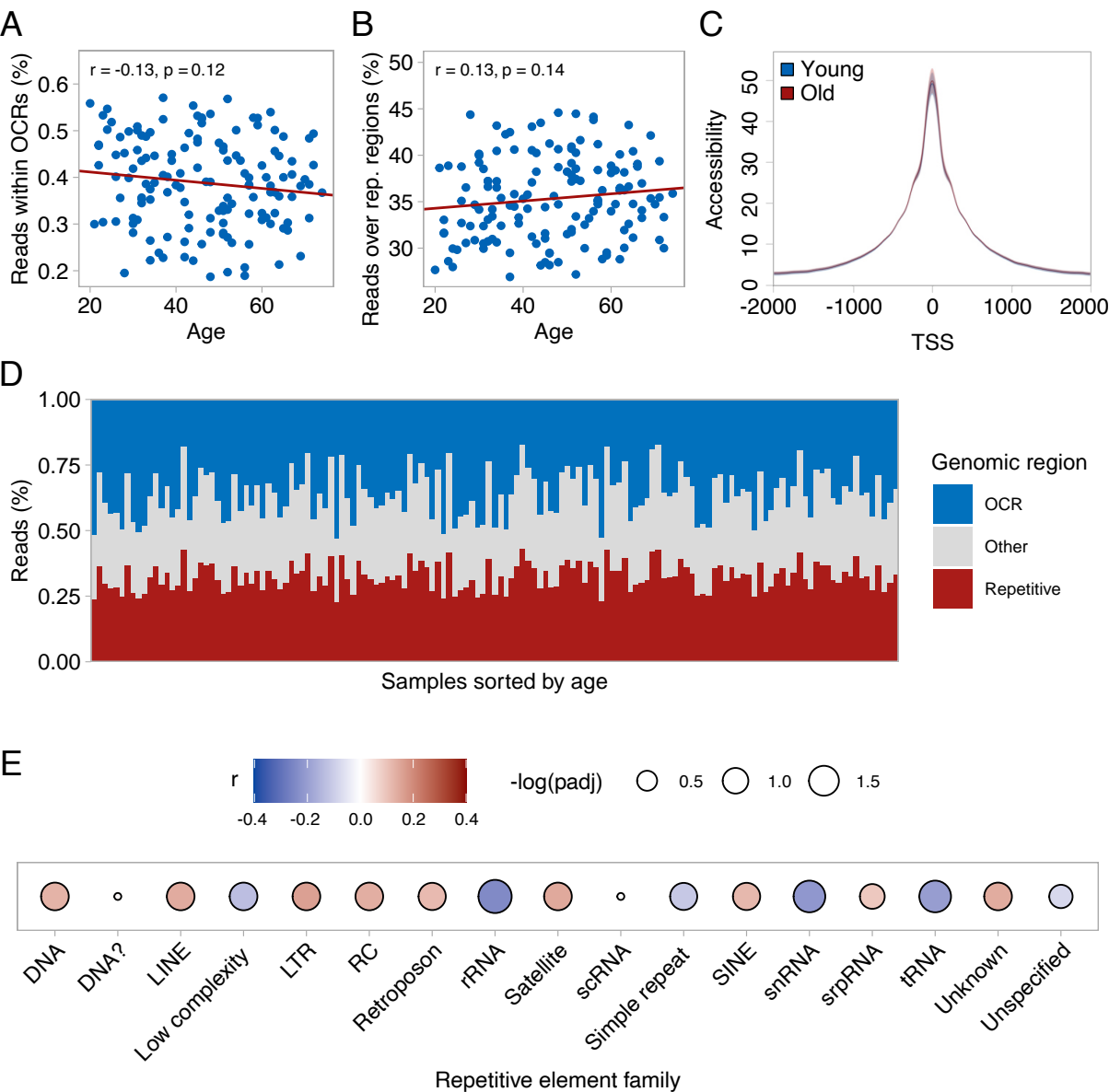

Supplement: Supplementary file 4 — Supplementary file4 (PDF 94 KB) [file 11357_2023_986_MOESM4_ESM.pdf]

Supplementary Figure 5

A

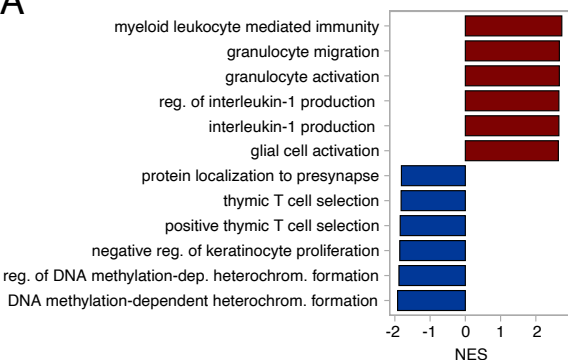

B

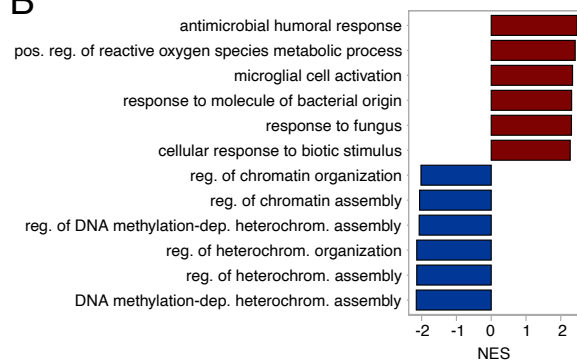

C

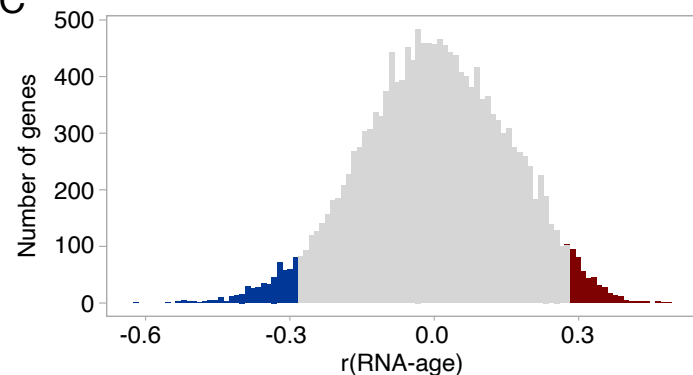

E

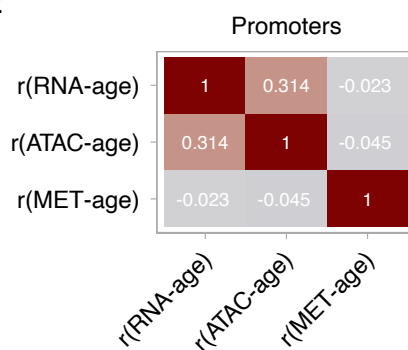

D

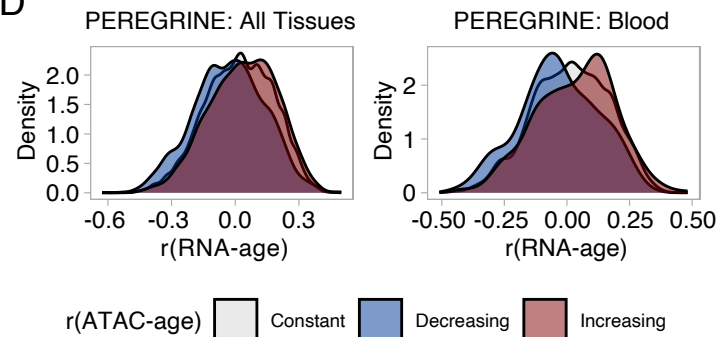

F

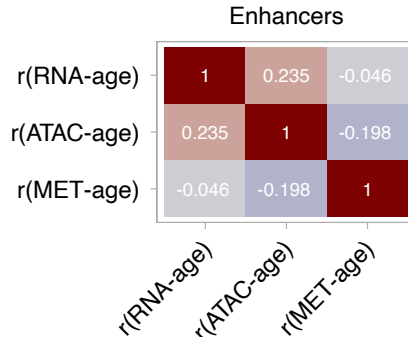

Supplement: Supplementary file 5 — Supplementary file5 (PDF 37 KB) [file 11357_2023_986_MOESM5_ESM.pdf]

Supplementary Figure 6

A

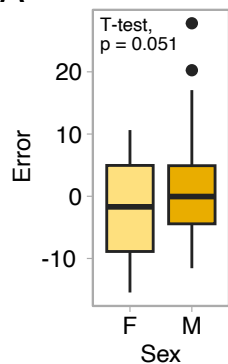

B

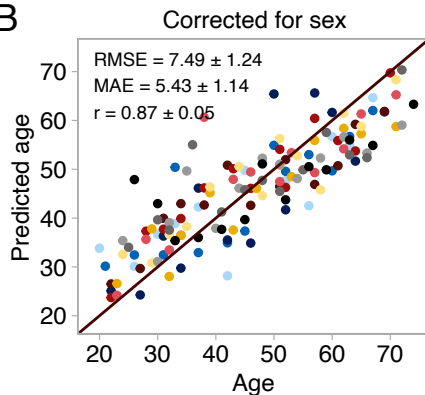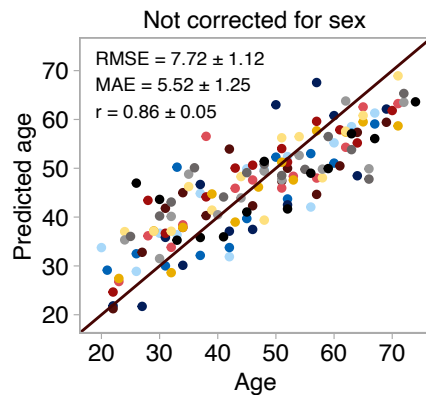

C

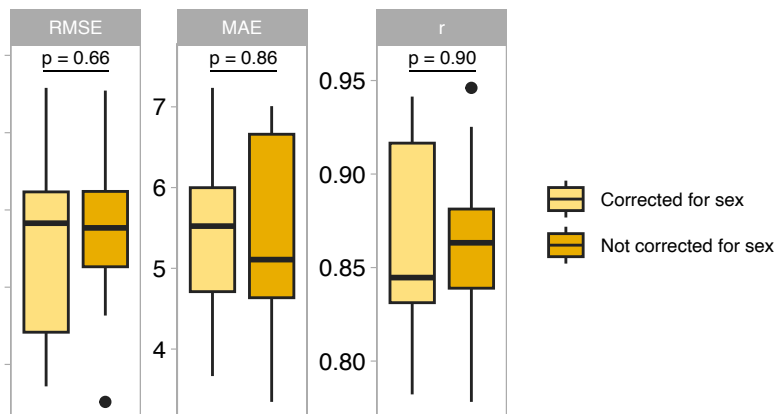

D

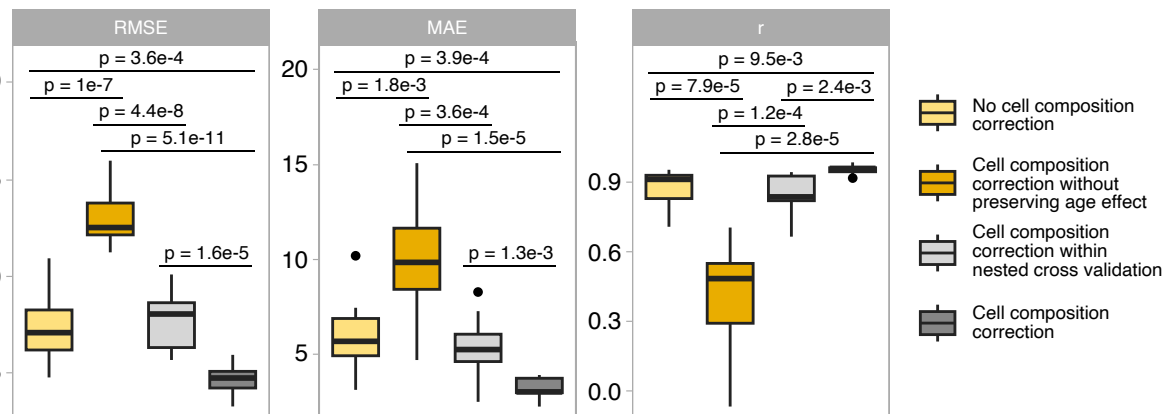

Supplement: Supplementary file 6 — Supplementary file6 (PDF 38 KB) [file 11357_2023_986_MOESM6_ESM.pdf]
